# Laparoscopic cholecystectomy in super elderly (> 90 years of age): safety and outcomes

**DOI:** 10.1007/s00464-023-10048-3

**Published:** 2023-04-24

**Authors:** Camilo Ramírez-Giraldo, Camila Rosas-Morales, Fiamma Vásquez, Andrés Isaza-Restrepo, Milcíades Ibáñez-Pinilla, Saul Vargas-Rubiano, Felipe Vargas-Barato

**Affiliations:** 1 Hospital Universitario Mayor - Méderi, Bogotá, Colombia; 2 Universidad del Rosario, Bogotá, Colombia

**Keywords:** Cholecystitis, Laparoscopic cholecystectomy, Elderly, Super elderly, Morbidity and mortality

## Abstract

**Background:**

Nonagenarian patients are an age group in progressive growth. In this age group, indications for surgical procedures, including cholecystectomy, will be increasingly frequent, as biliary pathology and its complications are frequent in this population group. The main objective of this study was to analyze the safety and outcomes of laparoscopic cholecystectomy in patients older than 90 years.

**Methods:**

A retrospective observational cohort study was designed. This study involved 600 patients that were classified in 4 age groups for analysis (under 50 years, 50–69 years, 70–89 years, and over 90 years). Demographic, clinical, paraclinics, surgical, and outcome variables were compared according to age group. A multivariate analysis, which included variables considered clinically relevant, was performed to identify factors associated with mortality and complications classified with the Clavien–Dindo scale.

**Results:**

The patients evaluated had a median age of 65.0 (IQR 34.0) years and there was a female predominance (61.8%). A higher complication rate, conversion rate, subtotal cholecystectomy rate, and prolonged hospital stay were found in nonagenarians. The overall mortality rate was 1.6%. Mortality in the age group over 90 years was 6.8%. Regression models showed that age over 90 years (RR 4.6 CI95% 1.07–20.13), presence of cholecystitis (RR 8.2 CI95% 1.29–51.81), and time from admission to cholecystectomy (RR 1.2 CI95% 1.10–1.40) were the variables that presented statistically significant differences as risk factors for mortality.

**Conclusion:**

Cholecystectomy in nonagenarian patients has a higher rate of complications, conversion rate, subtotal cholecystectomy rate, and mortality. Therefore, an adequate perioperative assessment is necessary to optimize comorbidities and improve outcomes. Also, it is important to know the greatest risk for informed consent and choose the surgical equipment and schedule of the procedure.

Nonagenarian patients, also known as super-elderly patients, are an age group in progressive growth [1]. According to the projections and retro projections of the national population in Colombia for the period 2018–2070, based on CNPV 2018, the population over 90 years of age represents 0.46% and is expected to reach 2.02% by the year 2070 [2], which for our population would represent 1,273,071 people, these projections are even greater for other latitudes [3]. In this age group, indications for surgical procedures, including cholecystectomy, will be increasingly frequent, as biliary pathology and its complications are frequent in this population group [3].

Some studies have identified a greater difficulty in laparoscopic cholecystectomy with increasing age, and although it is considered a safe procedure in elderly patients, it has been associated with greater technical difficulty, higher conversion rate, and increased complications of cholelithiasis associated with age [4–7]. The scales developed to predict the difficulty of cholecystectomy include age as one of its variables [8, 9]. In addition, the older the age, the greater the presence of multiple comorbidities and the reduction of functional reserves, and as a result performing surgical procedures on this group of patients is associated with a greater risk of complications [10].

Several studies have shown higher complications rates, conversion, and prolonged hospital stays as postoperative outcomes for groups of septuagenarian and octogenarian patients [6, 10–12], but few published studies have evaluated surgical outcomes in patients over 90 years of age.

The main objective of this study was to analyze the safety and outcomes of laparoscopic cholecystectomy in patients older than 90 years.

## Patients and methods

### Study design

A retrospective observational cohort study was designed. Between January 2014 and December 2021, 13.192 laparoscopic cholecystectomies were performed at the University Hospital Mayor and University Hospital Barrios Unidos—Mederi. Patients were classified in 4 age groups for analysis (under 50 years, 50–69 years, 70–89 years, and over 90 years) and a simple random sampling was performed until the sample calculated for each age group was reached. For the group over 90 years of age, all were included in the analysis because the calculated sample was not reached. The variables were collected in an anonymous database. This study was reviewed and approved by the ethics committee of the Universidad del Rosario (number DVO005 1904-CV1544). We followed the STROBE guidelines to report this study [13].

### Patients

Patients under 18 years of age; patients scheduled for open cholecystectomy; patients diagnosed with gallbladder cancer; patients whose cholecystectomy was associated with another surgical procedure (e.g., gastrectomy or pancreatoduodenectomy); and patients without postoperative follow-up or whose records did not have variables of interest were excluded.

Laparoscopic cholecystectomy was indicated in all cases by benign disease (biliary colic, pancreatitis, choledocholithiasis, cholecystitis, or the combination of these), and in all cases there was at least one image indicating biliary disease. In cases of acute cholecystitis, diagnosis, severity classification, and treatment were established according to Tokyo guidelines [14, 15]. Additionally, the American guidelines protocol was followed to identify the risk of choledocholithiasis: In low-risk cases, cholecystectomy was performed without further studies; in intermediate-risk cases, magnetic resonance cholangiopancreatography was performed; and in high-risk cases, endoscopic retrograde cholangiopancreatography (ERCP) was performed [16]. In cases of pancreatitis, cholecystectomy was defined when pancreatitis was clinically resolved.

All patients attended an outpatient monitoring appointment where the clinical evolution, surgical wounds, and histopathological outcome of the surgical specimen were reviewed.

We analyzed the following variables: Demographic characteristics of the patients; body mass index, ASA Physical Status Classification, presence of diabetes mellitus, arterial hypertension, chronic obstructive pulmonary disease, chronic kidney disease, cardiovascular disease, liver disease, the use of anticoagulants or antiplatelet agents, Charlson comorbidity index; preoperative laboratories and bile duct diameter on preoperative imaging; indication of surgical procedure; classification of severity of cholecystitis; preoperative ERCP; history of cholecystostomy; type of admission; time from admission to surgical procedure; preoperative risk scale for difficult laparoscopic cholecystectomy; intraoperative findings; conversion rate; type of cholecystectomy (total or subtotal); drain use; surgical time; complications associated with procedure and hospitalization; hospital stay; reintervention; and mortality.

The preoperative risk scale for difficult laparoscopic cholecystectomy evaluated was that described by Nassar, which includes factors related to laparoscopic cholecystectomy difficulty using the following preoperative criteria: age, ASA Physical Status Classification, primary diagnosis, thickening of gallbladder walls, preoperative ERCP, bile duct diameter, and type of admission. The scale used for intraoperative findings was the modified scale described by Nassar [9, 17] (Table 1).

**Table 1:** Intraoperative difficulty scale for laparoscopic cholecystectomy

Grade 1
Gallbladder—Floppy, non-adherent
Cystic pedicle—Thin and clear
Adhesions—Simple up to the neck/Hartmann’s pouch
Grade 2
Gallbladder—Mucocele, packed with stones
Cystic pedicle—Fat laden
Adhesions—Simple up to the body
Grade 3
Gallbladder—Deep fossa, acute cholecystitis, contracted, fibrosis, Hartmann’s adherent to common bile duct, impaction
Cystic pedicle—Abnormal anatomy or cystic duct—short, dilated, or obscured
Adhesions—Dense up to fundus; involving hepatic flexure or duodenum
Grade 4
Gallbladder—Completely obscured, empyema, gangrene, mass
Cystic pedicle—Impossible to clarify
Adhesions—Dense, fibrosis, wrapping the gallbladder, duodenum, or hepatic flexure difficult to separate
Grade 5
Mirizzi syndrome type 2 or higher, cholecysto-cutaneous, cholecysto-duodenal, or cholecysto-colonic fistula

### Surgical procedure

Laparoscopic cholecystectomy was performed using the standard 4-port technique in the American position. Dissection of the Calot triangle was performed until the critical safety window was reached, always performing dissection above the Rouviere’s sulcus and from lateral to medial. After reaching the critical view of safety, two proximal and one distal clip were placed in the cystic duct and the cystic artery separately and then cut between the clips and anterograde dissection of the gallbladder was performed. In cases where the critical view of safety was not reached, the surgeon decided at their discretion to perform a fundus first, subtotal cholecystectomy, or conversion to open procedure. In none of the cases was intraoperative cholangiography performed. It was also the surgeon’s discretionary decision to accommodate a drain on the surgical site.

### Statistical analysis

We calculated a sample of 206 patients for each age group with an expected complication rate of 8% for those over 90 years of age and a complication rate for those under 90 years of age of 2%, with a confidence interval of 95%, and a power of 80%.

Demographic, clinical, paraclinical, surgical, and outcome variables were described. The distribution was evaluated with the Shapiro–Wilk and Kolmogorov–Smirnov test and a non-normal distribution was found. Categorical variables were described as proportions and continuous variables as medians with their respective interquartile range (IQR). A bivariate analysis was performed, using the likelihood ratio chi-square test in the case of categorical variables and the Kruskal–Wallis test in the case of continuous variables to evaluate differences between the variables according to the previously established age groups, considering a statistically significant difference *p* < 0.05. Moreover, we calculated a *post hoc* multiple comparisons test in order to establish in which groups were differences found. A multivariate analysis, which included variables considered clinically relevant, was performed to identify factors associated with mortality and complications classified with the Clavien–Dindo scale.

The entire analysis was performed in SPSS®26, considering a statistically significant *p* < 0.05.

## Results

**Fig. 1:**
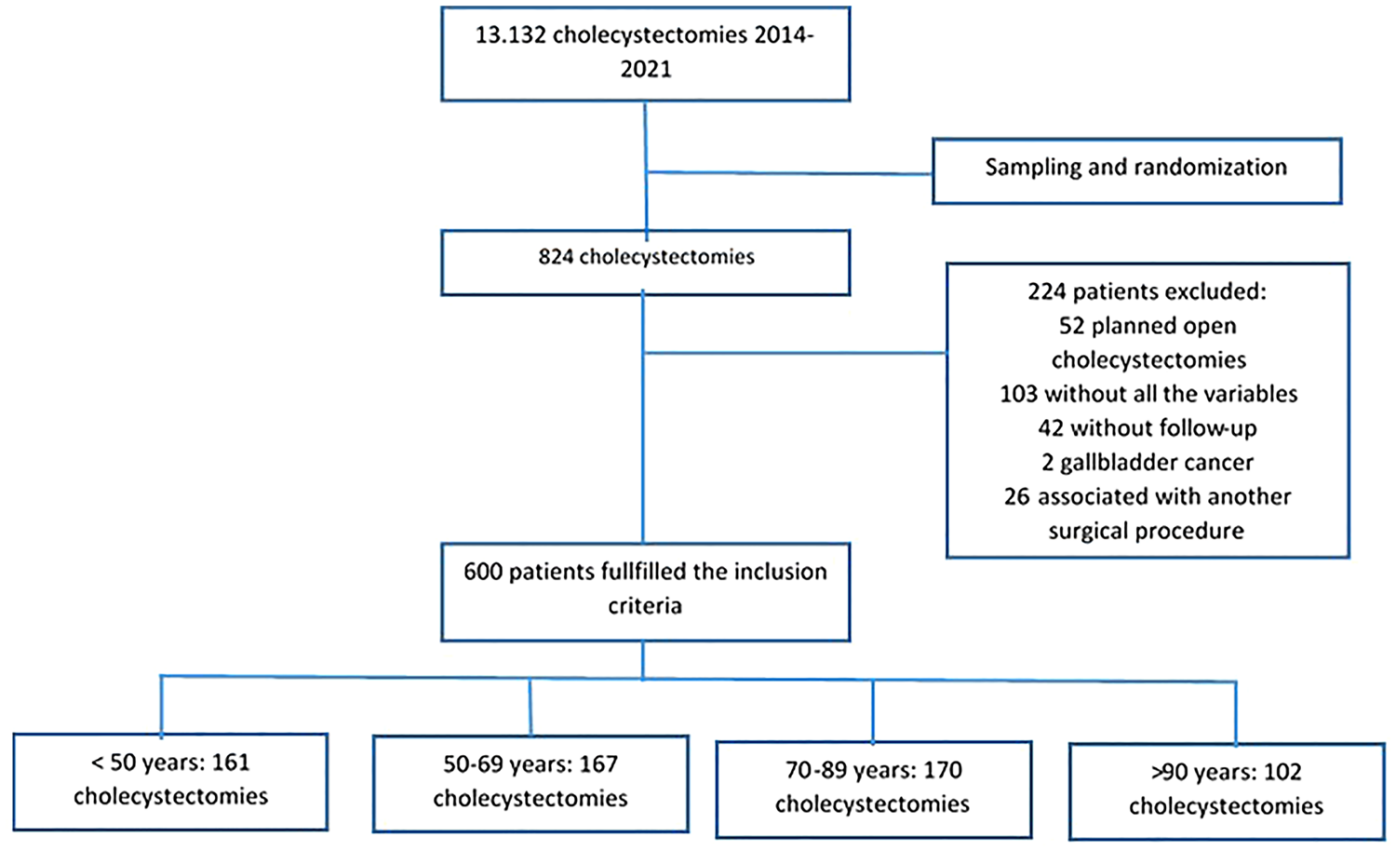
Flowchart of the study selection process

A total of 600 patients were included in the study, who were divided according to age groups into 161 under 50 years, 167 between 50 and 69 years, 170 between 70 and 89 years, and 102 over 90 years. The flowchart shows the selection process (Fig. 1).

The patients evaluated had a median age of 65.0 (IQR: 34.0) years and there was a female predominance (61.8%). In Table 2, the demographic, paraclinical, and clinical characteristics of the patients and the differences between these characteristics according to the age group are presented.

**Table 2:** Demographic, clinical, and surgical characteristics of patients with a cholecystectomy according to age group

	*N* (%)	< 50 years (%) *n* = 161	50–69 years (%) *n* = 167	70–89 years (%) *n* = 170	≥ 90 years (%) *n* = 102	Value *p*
Age (median)(IQR)(years)	65.0 (34.0)	36.0 (13.0)	59.0 (8.0)	76.0 (8.0)	92.0 (3.0)	
*Sex*						0.147
Female	371 (61.8)	105 (62.8)	108 (67.0)	104 (61.1)	54 (52.9)	
Male	229 (38.2)	62 (37.1)	53 (32.9)	66 (38.8)	48 (47.0)	
Body mass index (median)(IQR)(kg/m^2^)	25.7 (5.2)	25.8 (5.3)	26.6 (5.1)	25.6 (4.8)	23.4 (4.8)	< 0.001*^a^
*ASA classification*						** < 0.001**^a^
1	225 (37.5)	143 (85.6)	68 (42.2)	12 (7.0)	2 (1.9)	
2	241 (40.2)	23 (13.7)	79 (49.0)	105 (61.7)	34 (33.3)	
3	127 (21.2)	1 (0.6)	14 (8.7)	50 (29.4)	62 (60.7)	
4–5	7 (1.2)	0 (0.0)	0 (0.0)	3 (1.7)	4 (3.92)	
*Comorbidity*						
Diabetes mellitus	63 (10.5)	1 (0.6)	9 (5.5)	31 (18.7)	22 (21.5)	** < 0.001**^b^
Arterial hypertension	238 (39.7)	8 (4.79)	46 (28.5)	109 (64.1)	75 (73.5)	** < 0.001**^b^
Chronic obstructive pulmonary disease	72 (12)	2 (1.2)	9(5.5)	25 (14.7)	36 (35.2)	** < 0.001**^a^
Chronic kidney disease	27 (4.5)	0 (0.0)	1 (0.6)	12 (7.0)	14 (13.7)	** < 0.001**^b^
Cardiovascular disease	68 (11.3)	3 (1.8)	11 (6.8)	33 (19.4)	21 (20.5)	** < 0.001**^b^
Liver disease	7 (1.2)	4 (2.4)	0 (0.0)	0 (0.0)	3 (2.9)	**0.01**
Charlson comorbidity index (median)(IQR)(points)	3.0 (4.0)	0.0 (1.0)	3.0 (1.0)	5 (2.0)	6.0 (2.0)	< 0.001*^a^
Anticoagulants agents	23 (3.8)	1 (0.6)	4 (2.4)	14 (8.2)	4 (3.92)	**0.002**^e^
Antiplatelet agents	85 (14.2)	1 (0.6)	19 (11.8)	44 (25.8)	21 (20.5)	** < 0.001**^d^
*Preoperative laboratories (median)(IQR)*						
Leukocytes (× 10^3^)	11.3 (5.3)	9.9 (5.4)	10.6 (4.1)	11.0 (5.7)	12.0 (6.9)	0.008*^b^
Hemoglobin (mg/dL)	14.2 (2.7)	14.5 (2.6)	14.6 (2.4)	14.0 (3.0)	13.6 (2.5)	< 0.001*^b^
Bilirubins (mg/dL)	1.0 (1.1)	0.7 (0.8)	1.0 (0.8)	1 (1.1)	1.2 (1.7)	< 0.001*^b^
Alkaline phosphatase (mg/dL)	119.5 (91.5)	96.0 (74.0)	123.0 (70.5)	129 (91.7)	150 (194.7)	< 0.001*
Aspartate aminotransferase (mg/dL)	31.5 (69.0)	27 (65.5)	33.0 (56.0)	29 (53.7)	40 (157.5)	0.264*
Alanine aminotransferase (mg/dL)	39.0 (91.5)	37.0 (84.0)	40 (65.5)	31 (81.2)	54.5 (128.0)	0.145*
Bile duct diameter (median)(IQR)(mm)	4.0 (1.0)	4.0 (0.0)	4 (0.2)	4 (1.05)	4.2 (5.1)^γ^	< 0.001*^a^
*Indication of surgical procedure*						
Biliary colic	218 (36.3)	69 (41.3)	84 (52.1)	53 (31.1)	12 (11.7)	** < 0.001**^a^
Pancreatitis	74 (12.3)	16 (9.5)	15 (9.3)	28 (16.4)	15 (14.7)	0.128
Choledocholithiasis	87 (14.5)	16 (9.5)	15 (9.3)	24 (14.1)	32 (31.3)	** < 0.001**^a^
Acute cholecystitis	291 (48.5)	82 (49.1)	57 (35.4)	87 (51.1)	65 (63.7)	** < 0.001**^c^
*Classification of severity of cholecystitis*						** < 0.001**^a^
I	83 (13.8)	31 (18.5)	20 (12.4)	27 (15.8)	5 (4.9)	
II	140 (23.3)	49 (29.3)	29 (18.0)	41 (24.1)	21 (20.5)	
III	68 (11.3)	2 (1.2)	8 (4.97)	19 (11.1)	39 (38.2)	
*Preoperative ERCP*						** < 0.001**^a^
No	502 (83.6)	150 (89.8)	146 (90.6)	140 (82.35)	66 (61.7)	
Yes	98 (16.3)	17 (10.1)	15 (9.3)	30 (17.6)	36 (35.2)	
*Type of admission*						** < 0.001**^a^
Elective	121 (20.1)	37 (22.1)	42 (26.0)	37 (21.7)	5 (4.9)	
Delayed	466 (77.6)	129 (77.2)	118 (73.2)	129 (75.8)	90 (88.2)	
Emergency	13 (2.1)	1 (0.6)	1 (0.6)	4 (2.35)	7 (6.86)	
*History of cholecystostomy*						**0.004**^e^
No	592 (98.7)	167 (100.0)	161 (100.0)	164 (96.4)	100 (98.0)	
Yes	8 (1.3)	0 (0.0)	0 (0.0)	6 (3.5)	2 (1.9)	
Time from admission to surgical procedure (median)(IQR) (days)	3.0 (5.0)	2.0 (2.0)	2.0 (4.0)	4.0 (6.0)	6.0 (5.0)	< 0.001*^a^
Preoperative risk scale for difficult laparoscopic cholecystectomy (median)(IQR) (points)	6.0 (7.0)	5.0 (6.0)	3.0 (6.0)	7.0 (6.2)	9.0 (4.2)	< 0.001*^a^
*Intraoperative findings*						**0.003**^e^
1	165 (27.5)	60 (35.9)	38 (23.6)	38 (22.3)	29 (28.4)	
2	165 (27.5)	49 (29.3)	47 (29.1)	50 (29.4)	19 (18.6)	
3	113 (18.8)	31 (18.5)	31 (19.2)	38 (22.3)	13 (12.7)	
4	84 (14.0)	16 (9.5)	26 (16.1)	23 (13.5)	19 (18.6)	
5	73(12.1)	11 (6.5)	19 (11.8)	21 (12.3)	22 (21.5)	

The *p* values were obtained from the likelihood ratio Chi-square test

^*^The *p* values were obtained from the Kruskal–Wallis test

Bold values indicate statistically significant *p* values (*p* < 0.05)

^a^Age ≥ 90 years had a statistically significant difference compared to all other age groups

^b^Age ≥ 90 years had a statistically significant difference compared to all other age groups except for the age group 70–89 years

^c^Age ≥ 90 years had a statistically significant difference compared to age group 50–69 years

^d^Age ≥ 90 years had a statistically significant difference compared to age group < 50 years

^e^Did not present statistically significant differences when comparing the age group ≥ 90 years with the rest of the groups

We found that patients with an age ≥ 90 years had a statistically significant difference in the variables BMI, ASA classification, chronic obstructive pulmonary disease, Charlson comorbidity index, bile duct diameter, biliary colic, choledocholithiasis, preoperative ERCP, type of admission, time from admission to surgical procedure, and preoperative risk scale for difficult laparoscopic cholecystectomy compared to all other age groups. On the other hand, variables diabetes mellitus, arterial hypertension, chronic kidney disease, cardiovascular disease, leukocytes, hemoglobin, and bilirubin had statistically significant differences between the ≥ 90 years group age and all other age groups except for the age group 70–89 years. When comparing age group ≥ 90 years and the group < 50 years we found a statistically significant difference with antiplatelet therapy. Cholecystitis had a statistically significant difference comparing age group ≥ 90 years and age group 50–69 years. Anticoagulant therapy, intraoperative findings, and history of cholecystostomy did not present statistically significant differences when comparing the age group ≥ 90 years with the rest of the groups.

The overall mortality rate was 1.6%. Mortality in the age group over 90 years was 6.8%. The outcomes and their differences between the different age groups are presented in Table 3.

**Table 3:** Surgical outcomes following cholecystectomy according to age group

	*N* (%)	< 50 Years (%) *n* = 161	50–69 Years (%) *n* = 167	70–89 Years (%) *n* = 170	≥ 90 Years (%) *n* = 102	*p* value
*Conversion rate*						** < 0.001**^a^
No	569 (94.8)	161 (100)	156 (96.8)	156 (91.76)	90 (88.2)	
Yes	31 (5.17)	0 (0.0)	5 (3.1)	14 (8.24)	12 (11.7)	
*Type of cholecystectomy*						** < 0.001**^a^
Total	568 (94.6)	165 (98.8)	156 (98.8)	161 (94.7)	86 (84.3)	
Subtotal	32 (5.33)	2 (1.2)	5 (3.1)	9 (5.2)	16 (15.6)	
*Surgical time (median)(IQR)(minutes)*	73.0 (40.0)	70.0 (38.5)	70.0 (39.5)	80.0 (42.0)	77 (47.5)	0.250*
*Drain use*						** < 0.001**^a^
No	532 (88.6)	165 (98.8)	150 (93.1)	146 (85.8)	74 (72.5)	
Yes	68 (11.3)	2 (1.2)	11 (6.8)	24 (14.1)	28 (27.4)	
*Hospital stay (median)(IQR)(days)*	4.0 (7.0)	2.0 (4.0)	3.0 (5.0)	6.0 (7.0)	11.0 (7.5)	< 0.001*^a^
*Complications*						
Bile duct injury	11 (1.8)	2 (1.2)	1 (0.6)	1 (0.59)	7 (6.86)	**0.005**^a^
Bleeding	15 (2.5)	3 (1.8)	2 (1.2)	3 (1.76)	7 (6.86)	0.057
Intestinal injury	2 (0.3)	1 (0.6)	0 (0.0)	1 (0.59)	0 (0.0)	0.510
Surgical site infection	10 (1.6)	2 (1.2)	0 (0.0)	2 (1.18)	6 (5.8)	**0.005**^a^
Acute myocardial infarction perioperative	2 (0.3)	0 (0.0)	0 (0.0)	1 (0.59)	1 (0.9)	0.347
Pulmonary embolism perioperative	6 (1.0)	0 (0.0)	0 (0.0)	3 (1.76)	3 (2.9)	**0.019**^a^
Deep venous thrombosis perioperative	2 (0.3)	0 (0.0)	1 (0.6)	1 (0.59)	0 (0.0)	0.496
Health care-associated pneumonia	4 (0.6)	0 (0.0)	1 (0.6)	2 (1.18)	1 (0.9)	0.405
Health care-associated urinary tract infection	8 (1.3)	1 (0.6)	0 (0.0)	2 (1.18)	5 (4.9)	**0.011**^b^
Pleural effusion	12 (2.0)	3 (1.8)	1 (0.6)	3 (1.7)	5 (4.9)	0.110
*Reintervention*						**0.007**^a^
No	579 (96.5)	163 (98.2)	159 (98.7)	165 (97.0)	92 (90.2)	
Yes	21 (3.5)	4 (2.4)	2 (1.2)	5 (2.9)	10 (9.8)	
*Clavien–Dindo*						** < 0.001**^a^
I	34 (5.6)	6 (3.59)	2 (1.2)	13 (7.6)	13 (12.7)	
II	20 (3.3)	4 (2.4)	4 (2.4)	4 (2.3)	8 (7.8)	
IIIA	6 (1.0)	0 (0.0)	0.0 (0.0)	2 (1.1)	4 (3.9)	
IIIB	9 (1.5)	2 (1.2)	2 (1.2)	1 (0.5)	4 (3.9)	
IV	9 (1.5)	2 (1.2)	0 (0.0)	1 (0.5)	6 (5.8)	
V	10 (1.6)	0 (0.0)	0 (0.0)	3(1.7)	7(6.8)	
*Death*						** < 0.001**^a^
No	590 (98.3)	167 (100)	161 (100)	167 (98.2)	95 (93.1)	
Yes	10 (1.6)	0 (0.0)	0 (0.0)	3 (1.7)	7 (6.8)	

The *p* values were obtained from the likelihood ratio chi-square test

*The *p* values were obtained from the Kruskal–Wallis test

Bold values indicate statistically significant *p* values (*p* < 0.05)

^a^Age ≥ 90 years had a statistically significant difference compared to all other age groups

^b^Age ≥ 90 years had a statistically significant difference compared to all other age groups except for the age group 70–89 years

We found a statistically significant difference between the group age ≥ 90 years in comparison to the other groups in the following proportions: conversion to open procedure, subtotal cholecystectomies, drain usage, hospital stay, bile duct injury, pulmonary embolism, surgical site infection, complications according to Clavien–Dindo, reintervention, and mortality.

We performed two models, one of ordinal regression and the other a Cox regression with constant time to evaluate the risk factors associated with complications according to the Clavien–Dindo classification and mortality, respectively (Tables 4 and 5). Age, body mass index, ASA Physical Status Classification, Charlson comorbidity index, white blood cells, hemoglobin, bilirubin, alkaline phosphatase, bile duct diameter, diagnosis of cholecystitis, preoperative ERCP, type of admission, time from admission to cholecystectomy, and intraoperative findings in the models. The preoperative risk scale for difficult laparoscopic cholecystectomy was excluded from the model because it includes variables already found in it.

**Table 4:** Ordinal regression for complications according to Clavien–Dindo

	β_i_	CI95%	*p* value
Age ≥ 90 years	2.38	0.99–3.77	0.001
Acute cholecystitis	0.69	0.15–1.23	0.011
Longer time from admission to surgical procedure (days)	0.08	0.02–0.15	0.007

**Table 5:** Cox regression with constant time for mortality

	RR	IC95%	*p* value
Age ≥ 90 years	4.6	1.07–20.13	0.040
Acute cholecystitis	8.2	1.29–51.81	0.025
Longer time from admission to surgical procedure (days)	1.2	1.10–1.40	< 0.001

Collinearity was observed between age and Charlson comorbidity index, so the Charlson comorbidity index variable was excluded from the model, which is expected due to the higher age burden of comorbidities.

Regression models showed that age over 90 years, presence of acute cholecystitis, and time from admission to cholecystectomy were the variables that presented statistically significant differences as risk factors for mortality and complications.

The ordinal regression model and Cox regression with constant time were consistent in finding the same factors (age over 90 years, acute cholecystitis, and time from admission to cholecystectomy) that were associated with complications according to the Clavien–Dindo classification and mortality, respectively.

## Discussion

Acute cholecystitis is one of the most frequent causes of emergency consultation in hospitals. In general, the incidence of biliary disease is 19% in women and 10% in men, as is observed in our population where there is also a female predominance [1]. When not operated on, 25% of patients who consult for biliary colic will develop biliary complications such as pancreatitis, acute cholecystitis, or obstructive jaundice in the first year [1].

Elderly patients—and more often nonagenarians—who present with cholecystitis have a lower probability of spontaneous resolution of symptoms than younger patients, and an increased risk of gangrenous cholecystitis, biliary peritonitis, and choledocholithiasis [1]. The risk of recurrence in patients receiving non-operative management reaches 39% at 2 years [18] and failure to perform the surgical procedure during the initial hospitalization has been associated with worse survival at two years (hazard ratio 1.56, CI95% 1.47–1.65) [1]. For these reasons, urgent cholecystectomy is the preferred initial approach unless there are contraindications for the procedure.

Other published studies coincide with the findings in our study. A meta-analysis showed that with increasing age there was a significantly higher rate of global complications, major postoperative complications, conversion to open cholecystectomy, biliary leaks, postoperative mortality, and hospital stay [10]. A perioperative mortality risk was found to be 10 times greater in patients older than 80 years [10]. Our study also found that increasing age increased the conversion rate, complications, subtotal cholecystectomy rate, hospital stay, reintervention rate, and mortality. The use of drain was decided at the discretion of the surgeon, increasing its use with age, which is probably related to greater difficulty of the procedure.

In a series that included 22 nonagenarian patients who were compared with those under 90 years of age, a statistically significant difference in conversion rate and hospital stay was observed; however, it is a study limited by the small sample [3].

The mortality in nonagenarians reported in another study was 5.5% and in another series of patients older than 70 years mortality was 6%, both of which are similar to the rate found by us in our study [19, 20]. In another series, mortality rate in this age group reached 19.4% [21].

A study comparing the surgical results of cholecystectomies between octogenarians and nonagenarians showed a similar mortality rate, but with a higher rate of complications and a longer hospital stay in the nonagenarians [22].

Another study compared octogenarian patients presenting with cholecystitis according to their degree of severity, showing that patients with severe cholecystitis had a longer surgical time, needed an additional port insertion, and a longer hospital stay. In addition, patients with pulmonary comorbidity presented a higher risk of major complications [11]. Other studies have found that gangrenous cholecystitis and comorbidities such as diabetes, cerebrovascular events, chronic kidney disease, and lung disease are risk factors for a higher rate of postoperative complications [23]. In our patients, we can observe a collinearity between age and Charlson comorbidity index as risk factors for mortality and complications.

Furthermore, with an increase in age we can observe a decrease in the rate of elective surgeries and a longer time from admission to the performance of the procedure, which is probably related to the greater number of comorbidities and the perioperative management that these require.

Because of the higher rate of complications and mortality in elderly patients, which in many cases could be considered high risk, therapeutic alternatives such as non-operative management have been proposed. In cases of biliary colic or chronic cholecystitis, conservative management with diet and weight control may be considered [10]. However, later on the patient could be readmitted for cholecystitis, which would make the surgical procedure more difficult and would be related to a higher rate of complications [24].

Another option that could be considered in elderly patients would be the performance of a cholecystostomy, although there is increasing evidence suggesting the superiority of cholecystectomy to cholecystostomy, with a lower rate of major complications, hospital stay, and mortality [25–27]. In patients who underwent cholecystostomy, more than 25% were readmitted 30 days after discharge, in addition to a higher mortality rate, infection after the procedure, bleeding, and hospital stay compared with cholecystectomy [10]. In a clinical trial between laparoscopic cholecystectomy and cholecystostomy in patients with high risk defined as APACHE II greater than or equal to 7, similar mortality was found between the groups but with a higher rate of complications in the cholecystostomy group [28]. A meta-analysis to evaluate cholecystostomy outcomes in elderly patients found that cholecystostomy had a higher rate of mortality and readmissions than cholecystectomy [29].

Elderly patients often have gallstones for many years and therefore characteristics of chronic cholecystitis like obliteration of the planes in the Calot triangle and chronic fistulas such as Mirizzi syndrome; these are related to greater intraoperative difficulty evidenced by the intraoperative difficulty scale by Nassar we used. As we observe in our results, nonagenarians have more severe intraoperative findings, which has been related to unfavorable clinical outcomes [8–10, 30]. On the other hand, when we evaluate the risk of difficult cholecystectomy preoperatively, we also found a greater risk in nonagenarian patients which has been associated with a higher rate of bleeding, biliary leakage, conversion to open procedure, and failure to reach a critical view of safety [9].

Multidisciplinary assessment and proper optimization of comorbidities preoperatively with an experienced surgical team and a suitable technical team can help improve outcomes in nonagenarian patients [23]. When the decision for laparoscopic cholecystectomy is made, which is the treatment of choice for biliary pathology, we can resort to tools such as ACS-NSIQIP risk calculator that is validated and that can help us make decisions in this group of patients knowing probabilities of success and avoiding subjective judgments [31].

Within the limitations we found that this was a retrospective study and that the sample calculated for nonagenarian patients was not reached.

This study showed that laparoscopic cholecystectomy in the group of patients over 90 years of age is not an uncommon procedure in a general hospital today and is associated with a higher rate of morbidity and mortality. Additional studies comparing cholecystectomy and cholecystostomy in patients older than 90 years are needed to assess which one has better surgical outcomes.

## Conclusion

Cholecystectomy in nonagenarian patients has a higher rate of complications, conversion rate, subtotal cholecystectomy rate, and mortality. Therefore, an adequate perioperative assessment is necessary to optimize comorbidities and improve outcomes. Also, it is important to know the risks when signing informed consent, choosing the surgical equipment, and scheduling the procedure.

## Data Availability

Data are available on request through institutional review board of Hospital Universitario Mayor—Méderi. You can contact to request the data to jose.daza@mederi.com.co.
